# The Experiences Surrounding Romantic Relationships of Korean Bisexual Individuals

**DOI:** 10.3389/fpsyg.2023.1083446

**Published:** 2023-04-04

**Authors:** Jeongeun Park, Seojin Chung

**Affiliations:** ^1^Department of Counseling Psychology, Kyungil University, Gyeongsan, Republic of Korea; ^2^Ministry of National Defense, Seoul, Republic of Korea

**Keywords:** bisexual, qualitative research, South Korea, sexual minorities, romantic relationships

## Abstract

**Introduction:**

In recent years, the level of social and academic interest in sexual minorities has been gradually increasing. However, studies on bisexual individuals and the data on experiences of bisexual individuals outside the United States are scarce.

**Methods:**

To decrease the gap in the literature, this study examined the experiences of South Korean bisexual individuals in the context of romantic relationships via semi-structured, in-depth interviews. Participants were recruited through online-based platforms, and the interview with eight participants was analyzed.

**Results:**

Five main themes were identified: discovering myself as bisexual, being exposed to discrimination and exclusion, being affected by the sex of a romantic partner, protecting myself when engaging in a romantic relationship, and benefits of a romantic relationship.

**Discussion:**

The findings suggest that Korea’s heteronormative system and culture were the major challenges perceived by the participants. In conclusion, this study illustrates how sociocultural norms, social acceptance, and recognition affect bisexual identity in a romantic relationship.

## Introduction

Bisexuality has been defined in several ways (Bauer and Brennan, 2013; Flanders et al., 2017); several scholars used the term to describe individuals who are attracted to both men and women sexually and romantically (Halperin, 2009; Klesse, 2011; Cox et al., 2013). However, the definition is based on the binary notion of gender and does not reflect the expanded gender and sexual identities (Niki, 2018). Thus, recent studies on bisexuality (Flanders et al., 2017; Israel, 2018; Cipriano et al., 2022) suggest that the definition of “bi” in bisexual should be interpreted as being attracted to more than one gender. Studies on lesbian, gay, and bisexual individuals often treat lesbian/gay (hereinafter LG) and bisexual as a group, rather than considering them as separate groups (Feinstein et al., 2016b). However, accumulating research indicates that group differences exist, and the experiences of bisexual individuals are different from those of LG individuals (Balsam and Mohr, 2007; Hequembourg and Brallier, 2009; Brewster and Moradi, 2010). Bisexual individuals often encounter additional stigmas such as bisexual invisibility and discrimination from both the LG community and the straight community (Koh and Ross, 2006; Balsam and Mohr, 2007), and One of the areas in which such negative attitudes play a significant role toward bisexual individuals is romantic relationships (Armstrong and Reissing, 2014; Feinstein et al., 2016a,b).

### Bisexual identity and romantic relationships

Bisexual individuals face stigma and negative attitudes and beliefs from heterosexuals and LG, which is referred to as binegativity (Mark et al., 2020). Some of such beliefs regarding bisexuality are that bisexual individuals are immoral and cannot sustain a monogamous relationship (Mohr and Rochlen, 1999; Koh and Ross, 2006; Balsam and Mohr, 2007). Such a stereotype endorses the view that bisexual individuals are untrustworthy romantic partners (Mohr and Rochlen, 1999; Brewster and Moradi, 2010). Furthermore, bisexual identity is often considered unstable and a precursor to accepting the identity of LG (Balsam and Mohr, 2007; Friedman et al., 2014). Another research found that bisexual individuals face stigma from their partners that bisexual individuals are unfaithful, unreliable, and unstable and thus are unsuitable for romantic relationships (Israel and Mohr, 2004; Armstrong and Reissing, 2014; Feinstein et al., 2016a).

Research has documented heterosexual and LG individuals’ unwillingness to have a bisexual individual as a romantic partner. Ess et al. (2022) noted that almost one-third of LG individuals were hesitant to consider dating a bisexual individual. In addition, Armstrong and Reissing (2014) found that attitudes regarding involvement in romantic activities with a bisexual partner appeared to be more negative when the relationship being considered was more committed. Furthermore, Feinstein et al. (2014) examined heterosexual and LG individuals’ willingness to date bisexual individuals and found that they were less favorable toward engaging in romantic relationships with them than bisexual individuals. In sum, bisexual individuals who are open about their sexual orientation may face difficulties in finding a romantic partner (Feinstein et al., 2016a) due to the binegativity of many potential romantic partners.

Besides being considered unsuitable for a romantic relationship by others, romantic relationships themselves have impacted bisexual individuals in several ways (Feinstein et al., 2016b). For example, romantic relationships may worsen the invisibility of bisexual identity. Compared to LG, bisexual individuals may be perceived as heterosexual or LG based on the sex of their romantic partner, considering that people make assumptions about a person’s sexual orientation depending on the sex of their romantic partner (Hequembourg and Brallier, 2009; Ross et al., 2010). In other words, bisexual individuals may be able to either pass as heterosexual and have the appearance of having a majority status (Mark et al., 2020) or pass as LG and have the social status of a minority but not as bisexual unless they explicitly express their sexual orientation. Such experience causes bisexual individuals to experience uncertainty about their identity (Dyar et al., 2014). Taken together, bisexual individuals’ lives may become very different depending on whether they are in a romantic relationship and whether their romantic partner is same-sex or different sex, a circumstance that heterosexual and LG individuals do not experience.

Romantic relationship engagement can also be a significant factor in the mental health of bisexual individuals (Feinstein and Dyar, 2018). Molina et al. (2015) found that bisexual women who were in a relationship with a male partner tended to experience higher internalized binegativity and were more likely to experience symptoms of depression. The researchers argued that this tendency was due to greater exposure to binegative attitudes in the relationship. Feinstein et al. (2016b) concluded that relationship involvement was related to increased anxiety and assumed that increased anxiety might result from experiences of bisexual identity erasure.

### Korean context: Not LGBT-friendly place

South Korea (hereinafter Korea) has traditionally not been lesbiangay, bisexual, and transgender (hereinafter LGBT)-friendly for several reasons. Korean culture is deeply rooted in Confucianism, in which age, sex, and social status predetermine relationships with others. Since Confucian ideology values the traditional family with a husband, wife, and their biological children, homosexuality has been viewed as unnatural (Youn, 2018). In addition to Confucianism, Korean society has been influenced by conservative Christianity as well. Conservative Christian groups argue that homosexuality is immoral and abnormal, and this has led to anti-LGBT movements (Lee and Baek, 2017; Yi et al., 2017). Finally, Korea has been described as a homogenous country for a long time, and Korea has only recently started to become a multicultural society (Choi and La, 2019). Therefore, Korea has not been “LGBT-friendly” for a long time. In 2000, when Hong Seokchon revealed his sexual orientation, he became the first Korean celebrity to come out as gay but was immediately banned from the media industry due to his sexual orientation. Although the number of celebrities who support and advocate for sexual minorities publicly (e.g., BTS’ UN speech) has been increasing, no major celebrity has come out to the public until now except Hong Seokchon.

In the last 20 years, there has been an incremental change in societal attitudes toward sexual minorities in Korea (Youn, 2018), and LGBT social movements and communities have grown (Phillips and Yi, 2020). In addition, there has been a rapid increase in academic research and publishing on the topic of sexual minorities in Korea (Namkoong and Park, 2020). Despite such positive changes, Korea seems to have been struggling with social acceptance of sexual minorities, and legal protection for LGBT has not been achieved yet (Park-Kim et al., 2007; Yi and Phillips, 2015). According to the Korea Social Integration Survey in 2021 (Korea Institute of Public Administration, 2022), more than half of the respondents (54.1%) responded that they “do not accept” sexual minorities, which is alarmingly higher than other minority groups (e.g., 25.0% for North Korean defectors, 12.9% for foreign workers, 20.4% for people with disabilities). In addition, research on LGBT individuals in Korea suggested that experiences of social stigma and discrimination around LGBT are associated with low life satisfaction and suicide risk (Namkoong and Park, 2020). Moreover, same-sex couples do not have the right to marry in South Korea, whereas same-sex marriage is legal. In many parts of the world, including the United States and European countries. The fact that same-sex couples cannot be simply equal to different-sex couples may represent another form of homophobia (Walker, 2007). Furthermore, the enactment of the Comprehensive Anti-Discrimination Act, which can prevent discrimination based on sexual orientation, has been thwarted by conservative politicians and Christian groups who hold up the anti-gay movement (Lee, 2014).

Not only is there an unwelcoming environment for LGBT individuals overall, but also bisexuals have been more marginalized than other subgroups in the LGBT community. For example, the majority of well-established organizations or communities are only either for lesbians or gays (Kim and Choi-Kwon, 2021), which leaves bisexual individuals feeling isolated and feeling that it is hard to find social support. In several studies on Lesbian, Gay, and Bisexual (hereinafter LGB) in Korea, bisexual individuals appeared to have bi-specific stress and poorer mental health compared to LG (Kim and Choi-Kwon, 2021), which is consistent with findings from the research on LGB conducted in the United States (Koh and Ross, 2006; Bostwick et al., 2007). However, bisexuality has received little attention in Korean academia. To the best of our knowledge, there is no research that primarily focuses on bisexual individuals. Bisexual individuals are studied under the umbrella of LGBT research but never studied alone in Korea.

While research on the romantic experiences of bisexual individuals has been on the rise, most of such research has been carried out in the United States (DeCapua, 2017; Davids and Lundquist, 2018; Feinstein and Dyar, 2018). Thus, our research aims to contribute to the body of literature on the experiences of bisexual individuals outside the United States who have been underrepresented in academia. In particular, exploring the experiences of bisexual individuals in Korea, where social acceptance is low and political and legal rights for LGBT people are limited compared to the United States, may offer a picture of how culture and social norms affect the lives of bisexual individuals.

## Methods

A phenomenological approach was adopted in this study. As a phenomenological study focuses on capturing thick descriptions of people’s lived experiences, how they experience what they experience—how they perceive it, describe it, feel about it, and understand it (Polkinghorne, 1989; Moustakas, 1994; Creswell and Poth, 2016)—a phenomenological approach can help researchers gain an understanding of the essence of participants’ life experiences (Table 1).

**Table 1:** Participants demographics.

Pseudonym	Gender	Age	Relationship status at the interview	The partner’s gender at the interview
Jiwoo	Female	33	Married	Male
Jimin	Female	25	Single	
Subin	Female	25	In a relationship	Male
Soyoon	Female	28	Single	
Eunseo	Female	33	In a relationship	Male
Minjun	Male	29	Single	
Hajun	Male	29	In a relationship	Female
Yejin	Female	27	Single	

### Sample and recruitment

The participants were recruited through online LGBT communities and social media platforms. The criteria for inclusion in the study were as follows: (a) self-identified as bisexual, (b) cisgender, and (c) aged 19 years or older. Individuals who were interested in participating contacted the researchers by email or in an open-chat room offered by KakaoTalk, a mobile messenger app in Korea, to set up a time and date to meet to conduct the in-depth interview. Our study only targeted cisgender bisexuals as the transgender or gender non-conforming identified bisexual people may present unique experiences compared to cisgender bisexuals (Cashore and Tuason, 2009; Niki, 2018; Williams et al., 2020; Feinstein et al., 2022).

As the ideal sample size in qualitative studies has not been standardized, we referenced the existing literature on the sample size in phenomenological studies. Morse (2000) has suggested that six to ten participants would be adequate for phenomenological research, and Creswell and Poth (2016) suggested five to twenty-five participants. Thus, we intended to recruit approximately ten participants. Convenience sampling was adopted, and recruitment strategies included social media platforms, such as Twitter, contacting LGBT communities, and word of mouth. We recruited a total of eight participants, with six female and two male participants (Table 1). Participants’ ages ranged from 25 to 33, with a mean age of 28. One participant was married (legally), three participants were in a relationship, and the others (*n* = 4) were single. Two participants were open to all regarding their sexual orientation, the other two were open to some, and the others (*n* = 4) indicated that they were open to very few friends and no family members about their sexual orientation.

### Interview process

To gather data, we used semi-structured interviews. Semi-structured interviews use pre-established questions but also provide opportunities for open-ended questions. The questions included: “What were your experiences related to romantic relationships like?,” “What do romantic relationships mean to you?,” and “Have you ever had experiences related to romantic relationships just because you are bisexual?.” In addition, probes were used to gain more depth or additional information about participants’ responses. These were not prepared in advance; rather, they flowed from the participants’ responses. After the interviews, the recordings were transcribed verbatim and stored, so that they could be reviewed and analyzed afterwards.

Due to the COVID-19 pandemic, all interviews were conducted through Zoom, an online videoconferencing platform. Consent forms were given to and signed by the participants before the start of the interviews. The participants were informed that they could withdraw from the interview at any time, and the interview would be recorded. Interviews were 45–50 min on average but ranged from 40 to 75 min long.

### Positionality of researchers

Both researchers have previous research experience with sexual minority-related issues using a phenomenological approach and training experiences regarding sexual minority issues as well. Both researchers are familiar with both Korean culture and sexual minority issues. Prior to the data collection, we explored and identified our potential biases and assumptions (Hill et al., 1997; Hays and Singh, 2011) regarding bisexual individuals and their experiences related to romantic relationships. The first researcher stated that she believed that bisexual individuals would come out to same-sex partners at a higher rate compared to different-sex partners. The second researcher stated that she believed that bisexual individuals might find it difficult to come out to people in general but find it easy to come out to their partners.

### Data coding and analysis

The steps for analyzing the data for this study aligned with the recommended method by Colaizzi (1978), which proceeds as follows: (a) reading through the participants’ transcribed interviews several times in order to get a general feel for them, (b) identifying significant statements that pertain to the romantic experiences, (c) exploration of meanings, (d) organizing the collection of meaning into clusters of the theme, (e) writing a detailed a description of phenomena, (f) returning the findings of the study to the participants, and (g) making any necessary changes reflecting participants’ feedback. Finally, we included a doctoral student in the coding and analysis process who has extensive training experience related to sexual minorities to increase the validity of the analysis.

### Credibility

Multiple methods were utilized to increase the credibility of the data in our study: (a) Researcher bias was identified prior to the data collection (Creswell and Poth, 2016); (b) the data were consistently compared with the themes that developed in the study; (c) the results were checked with the participants (member-checking; Lincoln and Guba, 1986; Creswell and Miller, 2000), and (d) an external auditor, selected based on her extensive counseling experiences with the sexual minority population in Korea, examined and affirmed the themes and description of the themes.

## Results

We identified five themes of Korean bisexual individuals’ experiences regarding their identity in the context of romantic relationships: (a) discovering myself as bisexual, (b) being exposed to discrimination and exclusion, (c) being affected by the sex of a romantic partner, (d) protecting myself when engaging in a romantic relationship, and (e) benefits of romantic relationship.

### Discovering myself as bisexual

The majority of participants stated that they used to recognize themselves as heterosexual but reassessed that identification when they began to like someone of the same sex, after which they identified themselves as bisexual. Subin described her experience, “When I could not rule out the possibility of me having romantic feelings for women, I thought to myself ‘Oh, I might be bisexual,’ and accepted it.” Jiwoo added, “Dating helped me to establish my inclination.”

In addition, most participants explained that they did not have strong prejudice or resistance toward sexual minorities even before identifying themselves as bisexual. For example, Hajun said, “I’ve always had a pretty big interest in this subject (sexual minorities), so I left the door open.” The participants’ open and receptive attitude toward sexual minorities appears to have helped them explore the possibility of their own sexual identity as bisexual or sexual minority, hardly feeling repulsed.

However, participants stated that they had consistently censored their bisexual identity even after they identified themselves as bisexual, as they felt confused or uncertain about their identity. Especially they began to question whether they were still allowed to consider themselves bisexual after dating someone for an extended period, which did not allow them to feel attracted to or date someone of the other sex. For example, Jimin said, “I know I do not need approval for my own identity, but to call myself bisexual, I’m kind of under pressure like I should have pretty much the same proportion of inclination between liking both sexes.” Eunseo mentioned a similar concern. She stated:

Since I’m not dating women, I wonder if I can identify myself as bisexual. (ellipsis) Literally speaking, as if I proved it to myself, I used to think that it was something that needed to be proved to others. For example, let’s say that I have dated about 5 men and 7 women, and then I would not hesitate at all to say that I am bisexual.

Furthermore, after doubting and censoring their own identities, the participants concluded that there are no particular standards or requirements for being bisexual, and no one has a right to cast doubt as long as they identify themselves as bisexual. They also expressed their intention to dispute the social stereotype that one cannot be bisexual if they get married to or date someone of one sex for a long time.

### Being exposed to discrimination and exclusion

Participants referred to many incidents of discrimination or bias from romantic partners or potential romantic partners. Most of their experiences turned out to have some commonality. For example, the majority of female participants (*n* = 5) claimed they had heard about the disgust from the lesbian community, where bisexuals are likened to bats. A bat is an animal with a negative reputation in Korea. Bisexuals are described as opportunists who indecisively stick to one side or the other depending on the advantage they receive. Another commonality they mentioned was the prejudice existing in the lesbian community that bisexuals are treated as the ones “who will end up getting married to a man.” Eunseo said, “I once dated a lesbian, and she told me she hates bisexuals. She said, ‘bisexuals are just like a bat.’ I thought that kind of misconception had already gone away, but I found out that it still exists.” In other words, the meaning of likening someone to a bat relates to getting married to a man, which implies that bisexual individuals will eventually decide to have a marital relationship with a man if they want to stay inside the social stability or the comfort guaranteed by the system.

In addition, some female participants said that their boyfriends (heterosexual men) had treated them as sexual objects when they came out as bisexual. For example, Eunseo said, “When I told my boyfriend that I’m bisexual, he said to my face, ‘Alright. Then go date a woman and let the three of us have sex together’.” Yejin also described her experience of being treated as nothing more than a sexual object for the sole reason that she is bisexual. “On apps something like Tinder, when I made my bisexual identity open to all, a total stranger (heterosexual male) keeps texting me like, have you had a threesome or why do not you have a threesome with me and my girlfriend. I mean, I felt like I was being treated as a pornographic being, not as a normal human being.” Logically, participants said that the disgust people have toward bisexuality has made them feel lethargic or angry. Subin said “When I encounter that disgust, I feel sad. The first feeling I get is the sadness, but the second one is helplessness, as I wonder how much longer I have to kindly point it out and explain.”

### Being affected by the sex of a romantic partner

Most participants reported that they experienced the social meaning of passing for heterosexual or for gay/lesbian, living as bisexual. Specifically, they discovered the noticeable difference in the social recognition, social safety net, or social support system regarding their romantic partners depending on whether they pass for heterosexual (privileged), in other words, the social majority, or pass for gay/lesbian (under-privileged), as they experienced romantic relationships with both sexes.

Participants expressed that the fact that their romantic partner’s sex determines the contours of their own life used to arouse anger or sadness. Yejin said, “Why does my life keep changing like this, just depending on their sex? Why do I belong to a different system and culture each time? Why do I have to handle such unfair discrimination?” Subjin stated that when dating someone of a different sex, there was no social inequality and no obstacles for letting people know about their romance, whereas when dating someone of the same sex, they were actually frustrated by the fact that their romantic relationship had to be unknown to others and that any institutional protection, such as marriage, was not provided.

The person I’m dating is a heterosexual male. And though I’m not planning to marry him soon, we can imagine the future of us being together if we want to. One of the futures could be a marriage or something else, but anyway, if I marry him, I can be protected by the law. At the same time, the thought reminds me of my ex-girlfriend. While I was dating my ex-girlfriend, our relationship was not protected by the law just because she is the same sex as me, which makes me feel bitter. Just because my partner is a man, the law in this society protects me in many ways. But when the sex of my partner flips, that one difference suddenly stops protection. Whenever I notice that, it makes me frustrated.

Differences in social recognition, the presence of a safety net, or institutional support (considering that homosexual marriage is not legally recognized in Korea) based on sexual orientation were also one of the reasons that made it hard for the participants to completely affirm or accept their identity. Jiwoo, the only (legally) married female participant, stated that her marriage allowed her to feel safe because she knew that she could be perceived as “ordinary” and “normal” without her identity as bisexual being known to others.

Thinking that society might recognize me as an ordinary person without any issues, it really gives me a big comfort. And it also gives me a kind of confidence. I mean, I don’t want my orientation to be disclosed or known to others and, in a conclusion, I feel like I have the proof to show myself as a normal person by getting married. Even if anyone who knows my past romantic relationships points out something like “You used to like women,” I can say “Right, but I got married to a man.” This is like my shield or defense.

Participants also reported that they felt pressure to play the stereotypical gender roles that are taken for granted by society when engaged in different-sex relationships. In terms of the content of gender roles, a difference exists between female and male participants. For example, Jimin said, “I had to put up a makeup and dress up and let men spend more money, which is determined by society.” On the contrary, Minjun stated,

Even though my partner didn’t ask me to, I felt like I should behave according to what a society expects men to do. I felt compelled to act like a man like taking the lead. Especially, in terms of sexual intercourse, it seemed that I should be in the leading position, which was the opposite of what I wanted.

### Protecting myself when engaging In a romantic relationship

Most participants mentioned “bi-erasure,” which refers to the state of not being recognized as bisexual by others while dating someone. When they go out with someone of different sex, it is natural to pass for heterosexual. On the other hand, when they go out with someone of the same sex, they pass for gay/lesbian as if they were one of those groups. Subin gave the following description about the experience where her identity as bisexual just disappeared or was erased regardless of the sex of the person they are dating:

In a heterosexual community, it is so natural to recognize me as a heterosexual. They ask me questions like, “Are you going to marry him?” Without any doubt that I may not be heterosexual when I’ve been dating my boyfriend for pretty long. When I heard that kind of question, it got me thinking like, this is something that I would never hear if I were dating a girl. Hearing the majority of the questions makes me feel as if my identity as bisexual is erased by default. But the lesbian community and queer community are not the exception to not considering the possibility of me as bisexual.

Although participants expressed their discomfort about their bisexual identities being erased, it was not easy for them to come out due to bisexual phobia, either. Jimin said, “In my case, if people recognize me as heterosexual, I just tend to go with what they think because I feel like I cannot bear bi-phobia.” This tendency also applies to when the participants passed for gay/lesbian and heterosexual. Yejin stated, “I have passed for lesbian several times, but at this point, I just do not want to come out as bisexual, taking a risk of bisexual phobia, so I often pretend to be lesbian.”

All participants, but two participants who are open about their sexual orientation, shared their experiences of struggling between coming out to their boyfriend or girlfriend and choosing to hide their identity from them while dating. Some participants explained that the sex, sexual orientation, or attitude toward sexual minorities of their counterparts actually had an impact on the decision on whether to come out or not. As Subin said,

As for my first boyfriend, I met him at the gathering of the human rights movement. So, I was aware of what his values are and that’s why I felt okay to tell him. But in some cases, I couldn’t come out, because, you know, we can feel or read one’s thoughts while talking and I guess I unknowingly felt that he has a queerphobia.

Jimin stated that she had not shared her identity as bisexual or sexual minority until now because she believes it is safer not to let the romantic partner know. The participants’ previous experiences led them to set requirements about who would be the right person to date. Most of them turned out to be qualitative factors, such as the counterpart’s personality or attitude toward LGBTQ, rather than objective factors, such as partners’ sexual orientation or gender. It ultimately showed that participants have the desire to check whether the person is safe or if the person is willing to understand their sexual orientation and have a romantic relationship with them as they are. Soyoon explained, “Whether this person is safe or not is quite a big deal for me, so unless I can be sure that this person is definitely on my side, I do not think I can get into the official couple relationship.”

### Benefits of romantic relationship

Participants expressed that they have learned and grown in many respects through their relationship with their romantic partner, which is their most intimate relationship. Subin said,

I believe that I can live well alone. But as far as I’m concerned, I believe the romantic relationship brings the pleasure of doing something together, even if I can do it very well by myself. Like others, I think I’ve grown up little by little each time as I dated people.

Participants also stated that their dating experience helped them acknowledge themselves as bisexual, make an obvious identification, and feel more comfortable about accepting themselves as they are. They also expressed their gratitude for their previous dating experiences, which helped them accept and understand themselves as bisexuals, despite being well aware of the discrimination, prejudice, or difficulties that bisexuals face. Soyoon said, “When I look back on my past that I’ve once loved women as a bi-romantic, it still makes me feel good about that. So, I realized, I love me being bisexual.”

## Discussion

The goal of this study was to better understand the experiences of bisexual individuals in romantic relationships by using a phenomenological qualitative approach. Overall, we found several themes that captured the experiences of Korean bisexual individuals in several areas related to romantic relationships based on our analysis.

The findings of our study describe how bisexual individuals came to have an opportunity to develop their identity through the process of being attracted to someone who, due to their sex, they had not considered to be a potential romantic partner. Similar to the findings of studies that explored bisexual individuals’ identity development process (Weinberg et al., 1994; Brown, 2002), the majority of participants identified themselves as heterosexual in the first place, which is taken for granted by most people. As the participants acknowledged their same-sex attractions, they began to develop their identity as bisexual. However, some findings of our study are not consistent with the initial stage of the existing identity development model (Weinberg et al., 1994; Brown, 2002). For example, the participants did not report much confusion nor struggle regarding accepting same-sex attraction, which is surprising considering the non-LGBT-friendly climate in Korea. In addition, the majority of participants in our study identified themselves as bisexual after experiencing romantic attraction even when the romantic attraction did not end up in an actual romantic relationship, whereas Weinberg et al. (1994) suggested that sexual experiences with both sexes play a critical role in developing a bisexual identity. Although the inconsistency cannot be explained through the findings in our study, there are a couple of possible explanations. First, it has been 20 years since the identity model (Weinberg et al., 1994; Brown, 2002) developed, and attitudes toward sexual minorities and the social climate have changed dramatically during that period (Youn, 2018). As such, especially for younger generations, it may not be as hard to accept the possibility of being sexual minority. Second, as researchers who developed the identity development model argued, the identity development process may vary according to the specific conditions within the culture a person belongs to. Since identity development models for bisexuals (Weinberg et al., 1994; Brown, 2002) were proposed targeting the population in the United States, they may not fit the Korean population. Finally, some participants in our study shared that they were familiar with and interested in minority issues or the human rights movement even before they acknowledged their same-sex attraction. This may help decrease the confusion or discomfort with embracing same-sex attraction.

The contents of stereotypes and stigma around Korean bisexual individuals were different from the findings of other studies conducted outside of Korea. For example, some studies reported beliefs about bisexual individuals such as that they were attention-seeking (Flanders et al., 2016), LG in denial (Flanders et al., 2016), prone to infidelity (Flanders et al., 2016), sexual opportunists (Stroup et al., 2014), engaged in risky sexual activities (Herek, 2002; Kashubeck-West and Szymanski, 2008), had a greater possibility of carrying sexually transmitted infections (Herek, 2002; Klesse, 2011), or were promiscuous (Herek, 2002; Zivony and Saguy, 2018). However, in our study, “bisexual individuals are like bats” was the only stereotype that the majority of participants in our study mentioned. A bat is an animal that symbolizes an opportunist in Korean culture (Yoon, 2008), which means that bisexual individuals are considered to be opportunists. Based on what participants shared in our study, the definition of an opportunist in the context is that bisexual individuals would choose a different-sex relationship, get married, and enjoy the privilege of heterosexuality when they are ready to settle down. In other words, bisexual individuals are considered to be people who are capable of putting aside their same-sex attraction when they want to pass for heterosexual for their own safety in society, which was mentioned as one of the negative attitudes that LG communities hold toward bisexual individuals (Israel and Mohr, 2004).

The difference in the findings of our study and previous studies may be explained by the context where the conversation occurs. As people express their prejudice and stereotypes against an individual when the person is perceived as a member of an out-group or “other” (Herek, 2009), it is possible that bisexual individuals encounter a variety of stigmas or stereotypes from people who are not close to them. On the other hand, a romantic partner would not hold the same stereotype as others because they may have far more knowledge about their partner, which may explain why the participants in our study did not report much about bi-specific stereotypes.

Regarding why “bisexual individuals are like bats” was the most frequent stereotype, the social environment in Korea should be taken into account. In Korea, legal protection, including same-sex marriage legalization and social acceptance for sexual minorities, is not offered yet (Yi and Phillips, 2015) despite the continued efforts of the LGBT community. As such, the LG community may perceive the most striking difference between bisexual individuals and them as whether they are able to gain social support and legal protection. Compared to social recognition that cannot be accomplished on an individual level, other stereotypes about bisexual individuals include behavior on an individual level, which is why “bisexual individuals are bats” is the most common stereotype that Korean bisexual individuals face.

What participants in our study shared, how different their lives could be based on whether they could pass for heterosexual or not in Korea, provides a more detailed picture of how social support and acceptance differs based on whether a person fits a social norm or not. As made evident by some of our findings, the sex of a romantic partner shapes the way other people and society perceive the relationship. Everything was easy for the participants, including talking openly about the romantic relationship and planning the future when they were dating someone of different sex. However, it was the opposite when they were dating a same-sex person. The participants were able to witness how differently heterosexual-perceived relationships and homosexual-perceived relationships are treated by society clearly. Although only one participant in our study expressed that she knew that she would choose a different-sex relationship and get married to be safe despite her attraction to members of the same sex, it is plausible that bisexual individuals consider the cost of not conforming to the social norm when they make a dating decision. Wu et al. (2020) suggested that social consequences and punishment for violating a social norm may lead bisexual individuals to choose different-sex relationships over same-sex relationships, explaining the survey result of Pew Research Center (2013) that a majority of bisexual individuals (84%) were engaged in a different-sex relationship. Future research on whether and how social support, including legal protection, affects bisexual individuals’ dating decisions is warranted. However, it should be noted that even if bisexual individuals prefer engaging in different-sex relationships over same-sex relationships, the choice should not be used to strengthen prejudice regarding bisexuality. Rather, attention should be paid to how powerfully a social norm can control one’s life, even in personal areas such as attraction and romantic relationships.

However, the participants turned out to pay social normative relationship costs as well in exchange for acquiring social support. Specifically, the participants reported that they felt more pressure to conform to the traditional roles assigned to their gender when they were in a relationship with a member of different sex, which is consistent with a previous study that explored bisexual women’s experience in a mixed-orientation relationship (Kwok et al., 2020). Similar to the findings of Kwok et al. (2020), the female participants in our study stated that they were under pressure to conform to traditional femininity, such as wearing makeup, playing a passive role in dating, and being acceptive of men paying more when dating. Furthermore, bisexual men appeared to be no exception. A male participant also reported the experience of feeling similar pressure to female participants. However, the content was different because the gender roles that the male participants perceived to be assigned were different from those of the female participants. It has been well documented that in heterosexual relationships, gender norms powerfully shape intimate relationship expectations (Dworkin and O’Sullivan, 2005; Siegel and Meunier, 2019). However, the findings of our study demonstrate that such norms and expectations also apply to the relationship that bisexual individuals and their heterosexual partners are engaged in, which mirrors findings from Kwok et al. (2020).

### Limitations

The results of this study must be considered within the limitations of sampling. We attempted to recruit more participants by promoting the research through multiple channels such as personal connections, LGBT professional and student associations, online LGBT communities, and social media. Nevertheless, the recruitment of participants was challenging and ended with a sample of eight. We assume that the sample size of eight is small to represent Korean bisexual individuals, although the percentage of Korean adults who identify as LGBT has never been surveyed and remains unknown. Thus, the findings of this study should be considered exploratory and interpreted with caution.

In addition, the sample is biased in terms of age and marital status. All participants but one were in their twenties, and only one participant was married, which may limit the transferability of the findings to a national sample. For example, considering the median age at first marriage for both men and women in Korea is in their thirties, bisexual individuals in their thirties or older may think about the legal protection and social recognition that a heterosexual relationship can have in Korean society more than those in their twenties. We believe that a more diversified sample may have yielded different findings (specifically in terms of age and marital status). However, with the absence of communities for bisexual individuals in Korea, attaining the best sample was out of reach. Future research could emphasize greater diversity and enough size in the sample. Finally, our study only included cisgender bisexuals because we intend to avoid generalizing the findings to groups that may have different experiences from those of cisgender bisexual groups. However, future studies should focus on people who are transgender and bisexual to explore how gender identity intersects with sexuality.

## Data availability statement

The datasets presented in this article are not readily available because participants of this study did not give written consent for their data to be shared publicly. Requests regarding the datasets should be directed to the corresponding author.

## Ethics statement

Ethical review and approval was not required for the study on human participants in accordance with the local legislation and institutional requirements. The patients/participants provided their written informed consent to participate in this study.

## Author contributions

JP formulated the research idea, analyzed the data, took the lead in writing the manuscript, and supervised the final manuscript. SC performed data collection, analyzed the data, and contributed to writing the first manuscript. All authors contributed to the article and approved the submitted version.

## Conflict of interest

The authors declare that the research was conducted in the absence of any commercial or financial relationships that could be construed as a potential conflict of interest.

## Publisher’s note

All claims expressed in this article are solely those of the authors and do not necessarily represent those of their affiliated organizations, or those of the publisher, the editors and the reviewers. Any product that may be evaluated in this article, or claim that may be made by its manufacturer, is not guaranteed or endorsed by the publisher.
